# Reassessment of the Phylogenetics of Two Pygmy Grasshopper Generic Groups *Tetrix* and *Systolederus* through Mitochondrial Phylogenomics Using Four New Mitochondrial Genome Assemblies

**DOI:** 10.3390/insects15030174

**Published:** 2024-03-04

**Authors:** De-Long Guan, Chao-Mei Huang, Wei-An Deng

**Affiliations:** 1 Key Laboratory of Ecology of Rare and Endangered Species and Environmental Protection, Guangxi Normal University, Ministry of Education, Guilin 541006, China; 2023660006@hcnu.edu.cn (D.-L.G.); hcm2337244016@163.com (C.-M.H.); 2 School of Chemistry and Bioengineering, Hechi University, Hechi 546300, China; 3 Guangxi Key Laboratory of Rare and Endangered Animal Ecology, Guangxi Normal University, Guilin 541006, China

**Keywords:** mitochondrial genome, pygmy grasshoppers, Tetrigidae, phylogeny, taxonomic revision, divergence dating

## Abstract

**Simple Summary:**

Pygmy grasshoppers are a highly diverse group of insects that are distributed across China’s landscapes. Their small size, limited flight capabilities, and reliance on humid habitats make them an intriguing subject to study evolution and biodiversity patterns. This research focused on improving the phylogenetic classification system of pygmy grasshoppers, which relies heavily on physical traits that can sometimes be misleading. We sequenced the complete mitochondrial DNA of four pygmy grasshopper species to reconstruct the evolutionary relationships among these species. Comparative analyses of new and existing DNA data revealed conflicts regarding the breadth of two generic groups—*Systolederus* and *Tetrix*. The Systolederus generic group encompasses too much diversity, necessitating taxonomic divisions, while *Tetrix* diversity appears restricted by overly splitting similar species. Further assessments uncovered over 150 million years of pygmy grasshopper ancestry and evidence for rapid habitat colonization abilities in the *Tetrix* generic group. By integrating genetic data, we refined perspectives on the evolutionary affinities of these insects. This approach demonstrates the importance of coupling morphological and molecular techniques for robust biodiversity assessments amidst the intricacies of adaptation and convergence. Resolving such taxonomic limitations will strengthen future initiatives to document and preserve pygmy grasshopper diversity across changing landscapes.

**Abstract:**

Mitochondrial genomes offer pragmatic genetic markers to reconstruct evolutionary relationships and inform taxonomic classifications. Here, we present complete mitochondrial sequences for four Chinese pygmy grasshoppers (Tetrigidae), aiming to reevaluate phylogenetic patterns and morphological taxonomy. Our 17,643 bp, 16,274 bp, 15,086 bp, and 15,398 bp mitogenomes of *Exothotettix guangxiensis*, *Formosatettix longwangshanensis*, *Euparatettix sinufemoralis* and *Systolederus zhengi*, respectively, exhibit archetypal Tetrigidae architecture. We constructed phylogenies using 13 protein-coding loci from 39 Tetrigidae mitogenomes, revealing several genus-level clusters with statistically solid support, conflicts regarding *Ex. guangxiensis*, *F. longwangshanensis* merging into *Tetrix*, and two subclades of *Systolederus*. The dated divergence analysis indicates over 150 Mya of Tetrigidae ancestry, tracing the *Systolederus* generic group splits up to ~75 million years ago. Moreover, the *Tetrix* generic group radiated over 14 Mya across vast distributions, consistent with rapid adaptive dispersals. Our mitochondrial reconstructions suggest that *Synstolederus* is taxonomically overextended for a single genus, while the distinctiveness of *Ex. guangxiensis* and *F. longwangshanensis* from *Tetrix* appears questionable, and the *Tetrix* generic group comprises a potential tRNA-Ile coding region. Our integrative mitogenomic approaches will help resolve issues stemming from morphological taxonomy that is reliant on traits that are prone to convergence. This investigation enhances comprehension of Tetrigidae phylogeny and accentuates molecular systematics.

## 1. Introduction

Pygmy grasshoppers (Tetrigidae), colloquially termed wart-biters due to the characteristic diamond-shaped plates on their backs, represent a unique orthopteran family with widespread distribution across China’s landscape [1,2,3]. These diminutive insects exhibit limited flight capabilities and specialized feeding behaviors, subsisting primarily on mosses and decaying organic matter in humid habitats [4,5]. As generalized pioneers, pygmy grasshoppers demonstrate resilience in adapting to environmental pressures, undergoing adaptive radiation and subsequent speciation events over evolutionary timescales [6,7,8]. The Tetrigidae family encompasses over 1800 documented species, qualifying as one of the most speciose families within the Orthoptera order [1,9].

In light of Tetrigidae’s exceptional diversity, previous research efforts have focused extensively on elucidating systematic relationships within this group, relying predominantly on morphology-based classification approaches to construct robust taxonomic frameworks [9,10,11]. However, upon closer inspection of specific taxa, we were surprised to discover that some critical morphological traits used for classification may have been subjected to the overriding forces of adaptive evolution, such as convergent evolution or rapid adaptive radiations [7,8,12,13]. The influence of these processes could convolute interpretations of the species’ genuine phylogenetic affiliations. We directed our attention toward two notable tetrigid genera exhibiting such tendencies—*Systolederus* and *Tetrix*. Recently, *Systolederus* expanded to include the entire formerly recognized *Teredorus* genus based predominantly on a shared narrow fastigium and overlapping Asian distribution [14]. However, the researchers also noted that “this is a heterogeneous genus with currently 64 species in it, that has to be split into different genera in the future”. [14] This commentary casts uncertainty on the monophyly of *Systolederus* while indicating that the narrow fastigium and fastigial carinae shapes are nebulous features prone to evolutionary transformations. Alternatively, the model genus *Tetrix* is infamous for rampant taxonomic synonyms primarily attributed to the groups’ immense morphological variation, broad geographic ranges, and propensity to rapidly differentiate ecotypes with discrete wing polymorphisms across semi-isolated habitats [12,15,16,17]. These phenomena spur the inaccurate delineation of cryptic species or even novel genera among populations inhabiting disjoint geographic areas.

To mitigate such complications, we attempt to provide some phylogenetic clarity using a molecular-based approach, with complete mitochondrial genomes representing ideal tools for this endeavor. As the most ubiquitous genetic marker applied over the past two decades, mitochondrial genomes offer affordable sequencing, straightforward analyses, and ample nucleotide sites (over 10 kb) to construct phylogenies [18,19]. Hence, mitogenomic data constitute a pragmatic initial step for molecular-level systematic studies.

Accordingly, here we present complete mitochondrial sequences from four Chinese endemic species to enrich the existing repository of Tetrigidae genomes and evaluate issues surrounding *Systolederus* and *Tetrix*. Our focal species encompass *Exothotettix guangxiensis*, *Formosatettix longwangshanensis*, *Euparatettix sinufemoralis*, and *Systolederus zhengi*. By harnessing these mitogenomes, we strive to reshape perspectives on pygmy grasshopper evolution, date major divergence events, and accentuate the significance of this investigation to establish robust phylogenetic classifications. The analyses of *Ex*. *guangxiensis*, *F. longwangshanensis*, and *Eu. sinufemoralis* aim to corroborate their systematics. Our focal species’ morphological similarities with *Tetrix* could analogously reflect the possible convoluting impacts of *Tetrix*’s rapid adaptive radiations on conventional morphology-delimited taxonomy. Concurrently, *Systolederus zhengi* data are integral for reassessing the genus’s heterogeneous nature and tangled taxonomy to inform future efforts on rectification.

## 2. Materials and Methods

Collection and Genomic DNA Extraction of Samples:

*Ex. guangxiensis*, *Eu. sinufemoralis*, and *S. zhengi* were obtained from the wild, specifically from adult phenotypes within Guangxi, China. Likewise, *F. longwangshanensis* specimens were collected from adult sources within Longwanshan, Zhejiang Province. Following the species-level identification of each specimen, sample preservation occurred through complete immersion in ethanol (100%), and the samples were subsequently stored at −80 °C within the Guangxi Normal University repository. The extraction of genomic DNA was conducted from each specimen as a whole utilizing the Tiangen DNA kit (Tiangen Biotechnology, Beijing, China). This DNA was used later for sequencing operations through the Illumina HiSeq technology (Illumina, San Diego, CA, USA) at Novogene Co., Ltd. (Shanghai, China).

Mitogenomes Sequencing, Assembly, and Annotation:

Quotas of approximately 500 ng total genomic DNA underwent library preparation for 250 bp pair-end reads utilizing the NEBNext Modules (New England Biolabs, Ipswich, MA, USA). Complete mitogenome sequencing was performed bidirectionally using the comprehensive Illumina HiSeq X-ten platform for the whole-genome application. Raw reads of 300 bp were obtained for all four pygmy grasshoppers. The sequence data obtained from the four grasshopper species samples ranged in size from approximately 2.3 Gb for *Eu. Sinufemoralis*, 4.9 Gb for *Ex. guangxiensis*, and 2.25 Gb for *F. longwangshanensis*, to 6.5 GB for *S. zhengi*, the species that produced the most extensive dataset. After being quality-trimmed with Trimmomatic 0.38 [20], the clean data were put into the mitogenome assembly using the Getorganelle v1.7.5 software [20]. The complete mitogenome sequence of the type species for Tetrigidae, *Tetrix japonica* (Genbank ID: NC_018543), was used as the initial seed. The retrieved mitogenome assemblies were annotated using the MITOS Web Server (http://mitos2.bioinf.uni-leipzig.de/index.py, accessed on 21 September 2023) [21] and adjusted using other available Tetrigidae mitogenomes in the Geneious prime software. The annotated mitogenomic sequences have been deposited in GenBank (https://www.ncbi.nlm.nih.gov/genbank, accessed on 21 September 2023) with the accession numbers OR260076-OR260079. Detailed sequence information for each species is provided in Supplementary Table S1.

Data analysis:

The complete mitochondrial genome sequences of 35 Tetrigidae species, along with two outgroups (*Ellipes minuta*, NC_014488.1, and *Mirhipipteryx andensis*, NC_028065.1), were obtained from GenBank (Supplementary Table S2). *M. andensis* and *E. minuta* are two Tridactylids commonly used as outgroup taxa for phylogenetic analyses among Caelifera species of the infraorder Acrididea [22,23]. Along with our data, 13 complete protein-coding genes were extracted from the 40 sequences and concatenated into a single dataset using Phylosuite v1.2.2 [24]. Relative synonymous codon usage (RSCU) values were used to represent codon usage bias, and these values were calculated during the extraction process (Supplementary Table S3). The dataset was further processed using the embedded workflow within Phylosuit. The final alignment matrix was generated through MAFFT alignment v7 [25], and the single concatenated unit that was produced was put into Modelfinder [26] to find the best model, which is the “GTR + F + R10”. The maximum likelihood phylogenetic tree was constructed using IQ-TREE 2.2.2.3 [27], using the parameters of “--alrt 1000 -m MFP+MERGE --symtest-remove-bad --cmin 10 --cmax 60” [28]. The Bayesian inference phylogenetic tree was constructed using Phylobayes pb_mpi version 1.9, and the parameters were set as “-cat -gtr -x 10 1000 chain4” [29]. The divergence times were estimated using BEAST v.1.10.4 [30], and the calibration times estimated from previous studies were queried from the timetree.org website (http://timetree.org/, accessed on 15 October 2023). The nodes of *Tetrix* and *Alulatettix* (4.35~7.98 MYA), and *Tetrix* and *Trachytettix* (129.4~132.9 MYA), were selected for calibration. The parameters, including time priors and prior distributions, were set as “Yule Process” and “Normal”; the range was strictly set according to the queried calibration times. The uncorrelated relaxed clock model was selected with the relaxed distribution set as lognormal, and the same partition generated from the IQTREE was adopted. Finally, the MCMC generations and burn-ins were set as 10 million and 1000, respectively. The generated trees were imported into the Treeannotator to yield a consensus tree. Arcgis 10.7 was used to draw the distribution map.

## 3. Results

### 3.1. Four Newly Characterized Mitogenomes

**Figure 1:**
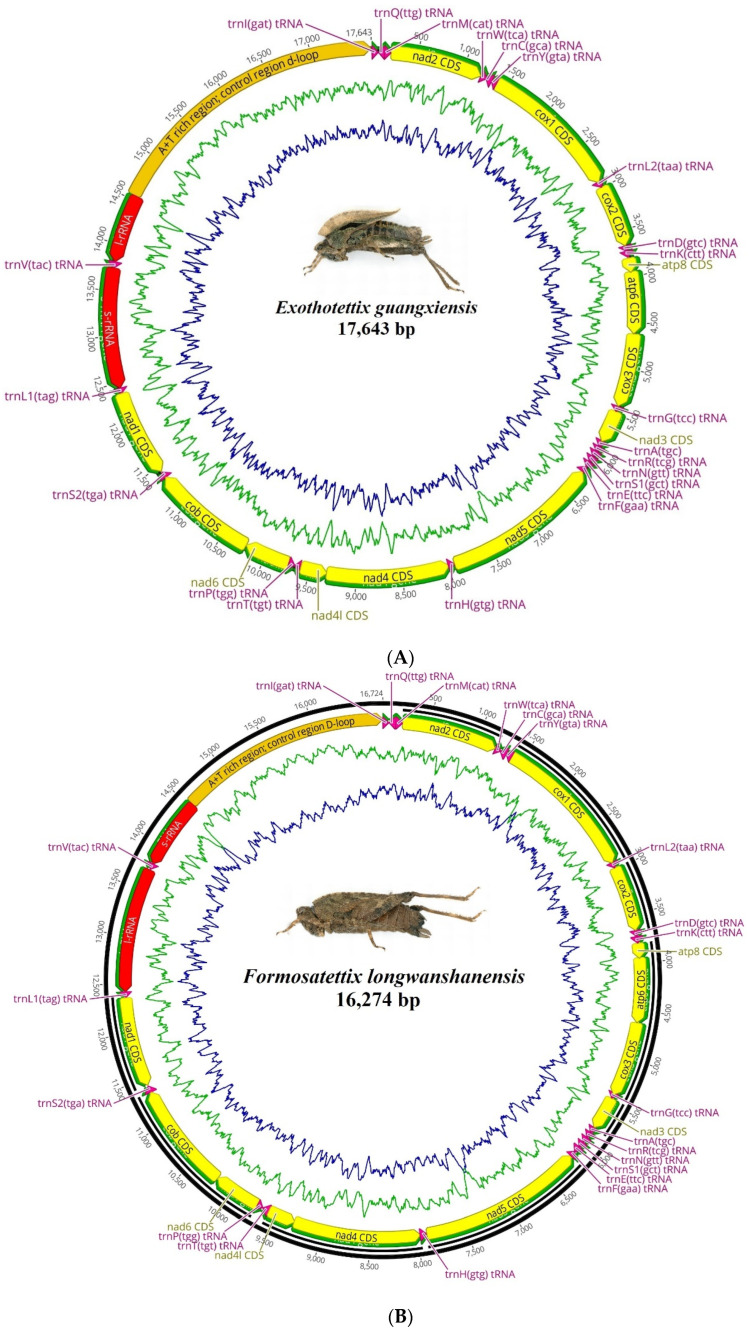

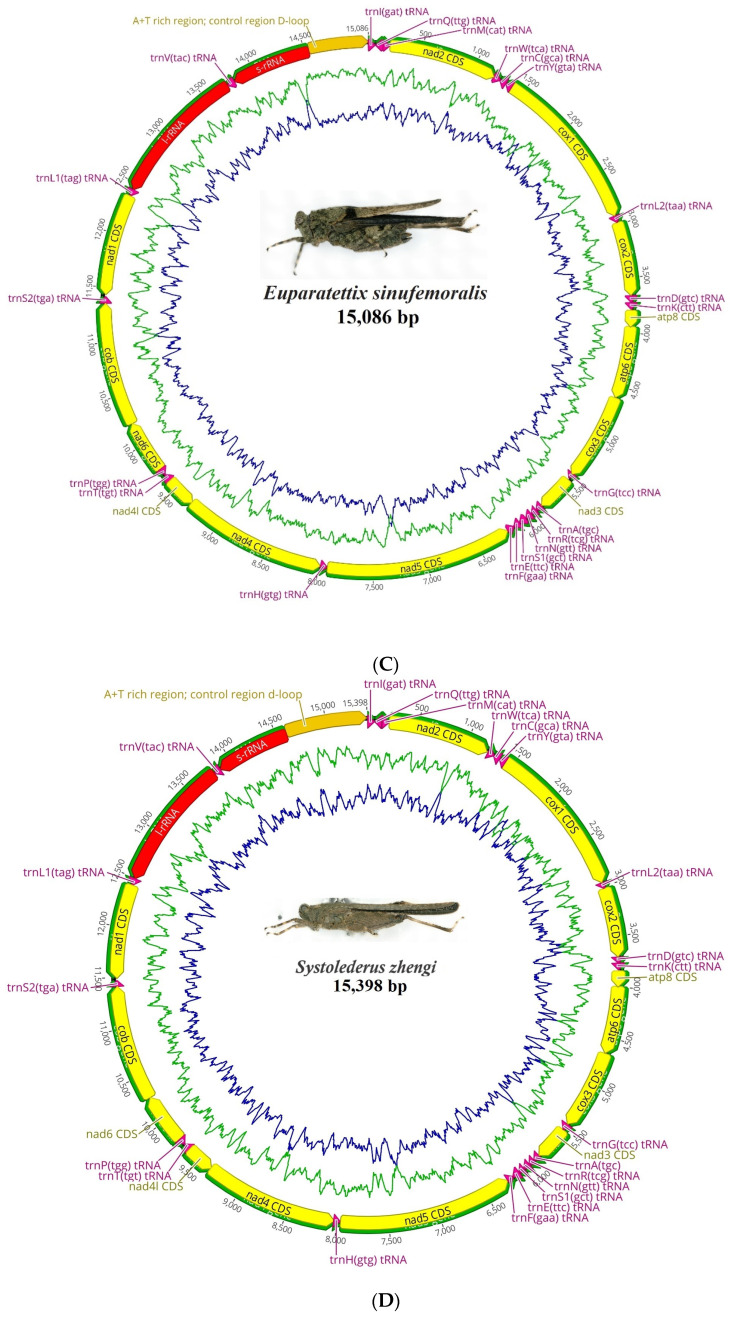
The circular view of the mitogenomes of *Ex. guangxiensis* (**A**), *F. longwangshanensis* (**B**), *Eu. sinufemoralis* (**C**), and *S. zhengi* (**D**). The yellow arrows represent the coding sequences (CDS), while tRNA genes are indicated by pink arrows, and rRNA genes by red arrows. The green and blue lines denote the AT and GC base content using a 100 bp sliding window approach.

The assembled mitochondrial genomes (mitogenomes) for *Ex. guangxiensis*, *F. longwangshanensis*, *Eu. sinufemoralis*, and *S. zhengi* exhibit lengths varying from 15,086 bp to 17,643 bp, as detailed in Figure 1. These assembled mitogenomes exhibit remarkable conservation, mirroring the characteristics prevalent in the pygmy grasshopper clade; they manifest not only comparability in length and gene content, but they are also homologous regarding gene arrangement and base usage bias. Presently, the scale of the Tetrigidae mitogenome lengths that have been made accessible ranges from 14,247 bp (*Criotettix japonicus*, Genbank ID: MT162542.1) to 17,859 bp (*Thoradonta yunnana*, Genbank ID: NC_071832.1). All the recently assembled mitogenomes of this study fall within this spectrum. The compact mitogenome of *Systolederus zhengi* comes in at a minimum length of 15,398 bp, whereas that of *Ex. guangxiensis* exhibits the maximum count, reaching up to 17,643 bp. An extension in the non-coding region is observed for the latter.

**Figure 2:**
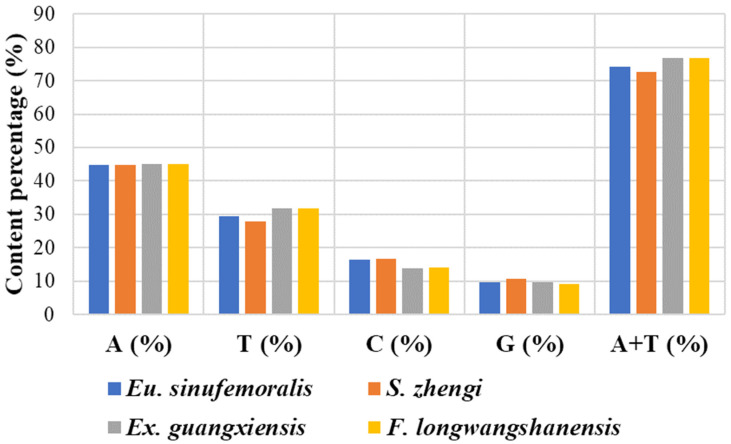
The base compositions for our newly obtained four mitogenomes.

Each of the consolidated mitogenomes from these four pygmy grasshoppers is identified to comprise 37 characterized mitochondrial genes. These include 13 protein-coding genes (PCGs), 22 transfer RNAs (tRNAs), two ribosomal RNA (rRNA) genes, and a lone non-coding region encroaching the 200 bp mark. This gene layout is similar to that observed in all pygmy grasshoppers, with 9 PCGs and 14 tRNAs located on the major strand and the remaining entities, encompassing both rRNAs, on the minor strand. Mutation tendencies within the domain of pygmy grasshoppers skew towards the AT duplex bond, substantially biasing the nucleotide composition in favor of A and T, resulting in an overarching AT content exceeding 70%. Predominantly, A usage outstrips that of T. In contrast, C usage surpasses G, rendering A as the most favored base and G as the least (Figure 2). In the mitogenomes of pygmy grasshoppers, the most A + T-rich regions correspond to the extended non-coding areas, which are believed to conceal the putative origin of mitogenome replication.

### 3.2. Conserved Mitochondrial tRNA-Ile Coding Region

The conservation of mitochondrial gene arrangement is a crucial indicator of species evolution and taxonomic boundaries. Typically, mitochondrial genomes are characterized by a tight packing of genes, often with little to no non-coding gaps between them. However, aberrant gene gaps exceeding 20 base pairs (bp) have been consistently reported between the tRNA-ser and nd1 genes in almost all Tetrigidae mitogenomes. Unlike the typically compact mitochondrial gene layouts, these gaps prompted a re-examination of the potential coding capability within these interspersed regions in the mitochondrial genomes of known Tetrigidae species.

**Figure 3:**
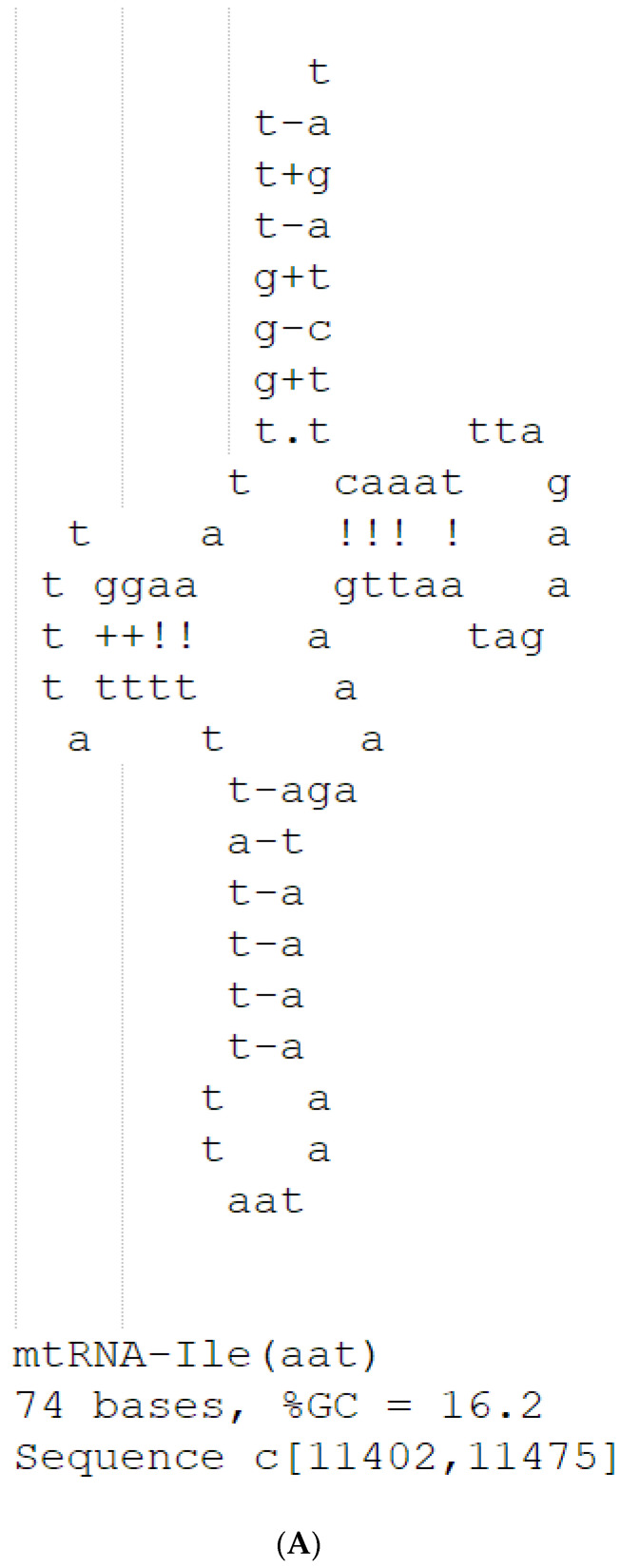

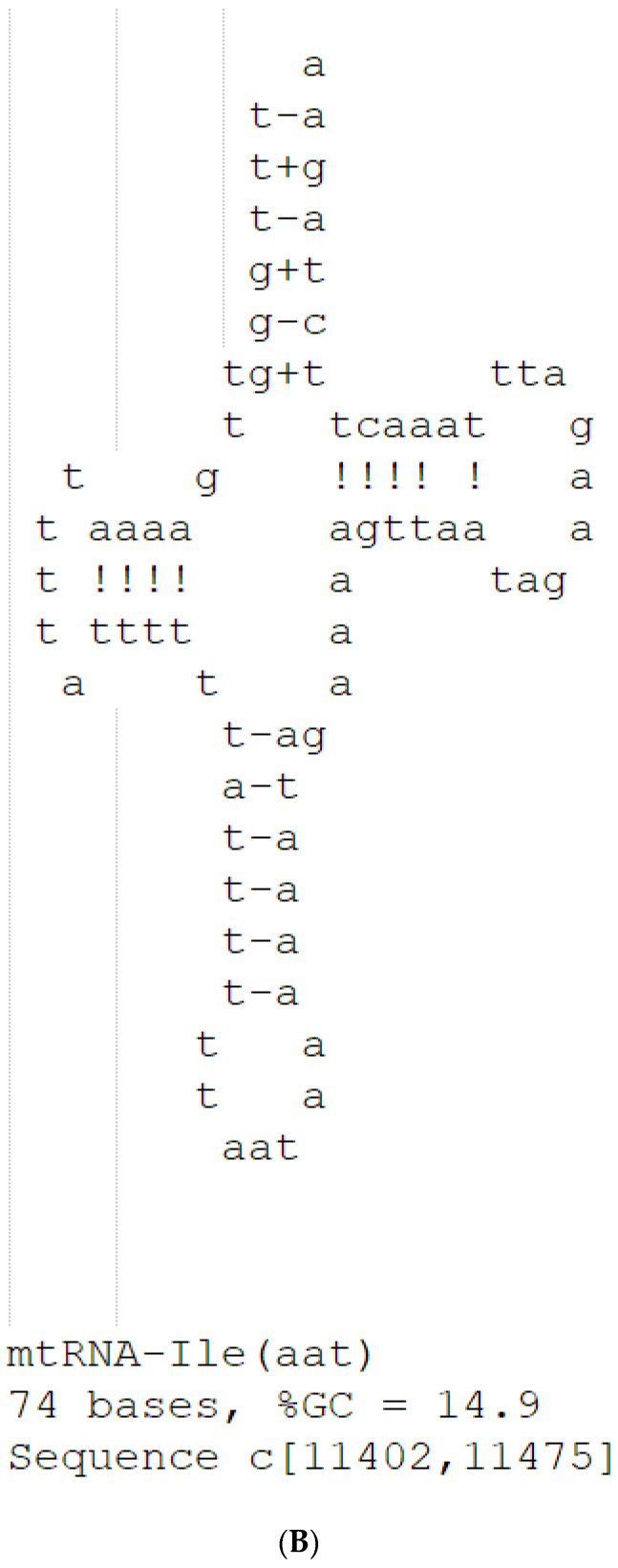
The sequence and predicted cloverleaf secondary structure of the potential tRNA-Ile are identified in the gap of these selected Tetrigidae mitogenomes. (**A**) *Ex. Guangxiensis,* (**B**) *F. longwangshanensis*, *A. yunnanensis*, *T. japonica*, and *T. ruyuanensis*.

Contrary to what might have been anticipated from the invariable nature of mitochondrial gene order in these insects, we have identified a novel, highly conserved potential tRNA-Ile (anticodon: aat) coding region within the said genetic gap in five different species, including *Ex. guangxiensis*, *F. longwangshanensis*, *A. yunnanensis*, *T. japonica*, and *T. ruyuanensis*. The putative gene spans 74 base pairs and is predicted to fold into a typical cloverleaf secondary structure, as illustrated in Figure 3A,B. This conserved tRNA-Ile sequence could explain the longstanding enigma of the genetic gap observed in the Tetrigidae mitogenome, suggesting that the insert or loss of this tRNA rearrangement may account for the gaps. Furthermore, the uniformity of this tRNA sequence across examined species points to a close evolutionary relationship, likely deriving from a singular evolutionary event. The conservation of this gene region brings the possibility that the current taxonomical division into these distinct genera within the family might require reassessment. The implications of this finding provide a compelling case for reclassifying these taxa, potentially amalgamating them under a single genus due to the shared novel mitochondrial feature, further reinforcing the value of mitochondrial DNA as a tool for phylogenetic studies.

### 3.3. Codon Usage Patterns in the Mitogenomes of Pygmy Grasshoppers

The analysis of the codon usage patterns in four closely related species, *Eu. sinufemoralis*, *Ex. guangxiensis*, *F. longwangshanensis*, and *S. zhengi*, revealed intriguing insights into the interplay of mutation bias, natural selection, and genetic drift in shaping codon usage. Despite their close evolutionary relationship, these species exhibited minor protein-coding gene (PCG) length variations, ranging from 11,092 bp in *Eu. sinufemoralis* to 11,117 bp in *S. zhengi*. These variations hinted at the influence of disparate evolutionary pressures on codon usage patterns among the species.

Notably, the species exhibited distinct codon usage patterns, suggesting that they experienced unique selective pressures during their evolutionary histories. These variations in codon usage patterns provide the initial evidence supporting the proposition of disparate evolutionary pressures among the species. The observed variations in codon usage patterns among the four species underscore the complex interplay of evolutionary forces that have shaped their genetic makeup. These findings contribute to our understanding of the molecular evolution of these species and provide a foundation for future studies investigating the specific selective pressures that have influenced their codon usage patterns. 

**Figure 4:**
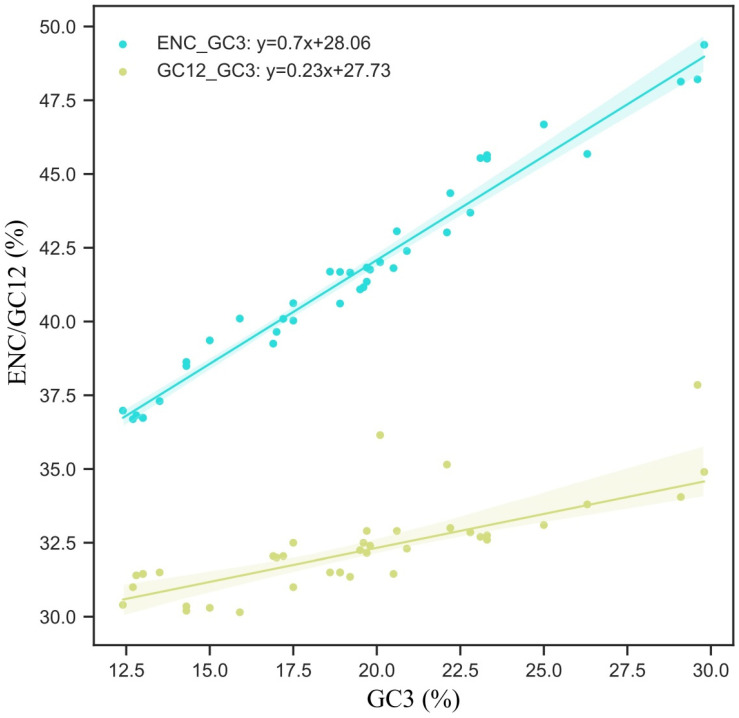
The regression analysis of ENC and GC12 with GC3. The blue dots represent the correlation between ENC and GC3, while the yellow dots depict the correlation between GC12 and GC3. The predicted regression equations are provided in the top-left corner of the figure.

In our initial examination of the general pattern, we computed the Effective Number of Codons (ENC)—an index capturing the multifaceted aspect of codon usage. Simultaneously, we estimated correlations between the GC density at the initial and secondary codon positions (GC12) and the third sequential codon location (GC3). Our investigations unearthed robust associations between ENC/GC12 and GC3, with nearly the totality of the data corpus positioned within the 95% confidence interval (Figure 4). Such findings serve as compelling evidence that, within each species, the degree of the mutational skew has a dominant influence on the configuration of the PCG codon inclination patterns. Further, they suggest that the relative impacts of erratic natural selection pressures across a multiplicity of species are relatively marginal. The derived regression model delineating the association between GC12 and GC3 reads as y = 0.23x + 27.73, boasting a slope of 0.23 (Figure 4). Intriguingly, this is substantially less than unity, alluding to a discernible bias in mutation directionality. Such a result underscores a distinct proclivity for substitutions from high-energy GC bonds to ones with lower energy, specifically, AT bonds.

**Figure 5:**
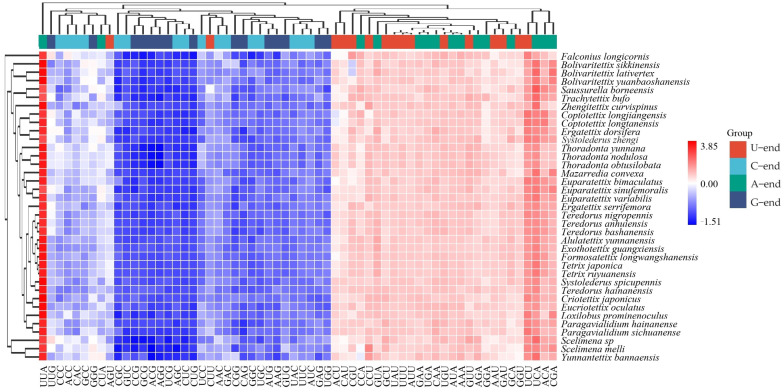
Heatmap of the RSCU values of all PCGs within the mitogenomes of the insects of the Tetrigidae family. Both the rows and the columns were clustered.

Then, we measured and compared the Relative Synonymous Codon Usage (RSCU) values for the species used in the further phylogenetic construction and subsequently illustrated them within a heatmap (Figure 5). We noticed that the codon clusters broadly bifurcated into two primary categories. The unfavorable codons (those with RSCU values < 1) grouped on the left side of the heatmap, whereas the favored codons (those with RSCU values > 1) populated the right. The UUA codon emerged as the most prevalent, while the ACG codon stood out as the least utilized. Impressively, all codons demonstrated a preference for ending with AU, reflecting the overall nucleotide makeup of PCGs and giving credence to the potent influence of directed mutation in shaping this pattern.

Focusing on specific species, we noticed that *Ex. guangxiensis* and *F. longwangshanensis* demonstrated significant similarities in their codon bias with *Tetrix* (Figure 5). This indicates that these species underwent similar evolutionary pressure patterns during differentiation, suggesting that they are closely related species with possibly short divergence times. In stark contrast, the relationships among the *Systolederus* generic group are chaotic (Figure 5), and we conclude based on this that *Systolederus* is indeed a genus with complex evolutionary relationships, where the mechanisms of differentiation and speciation may differ significantly even for species currently placed within the same genus.

### 3.4. Mitochondrial Phylogenomics

**Figure 6:**
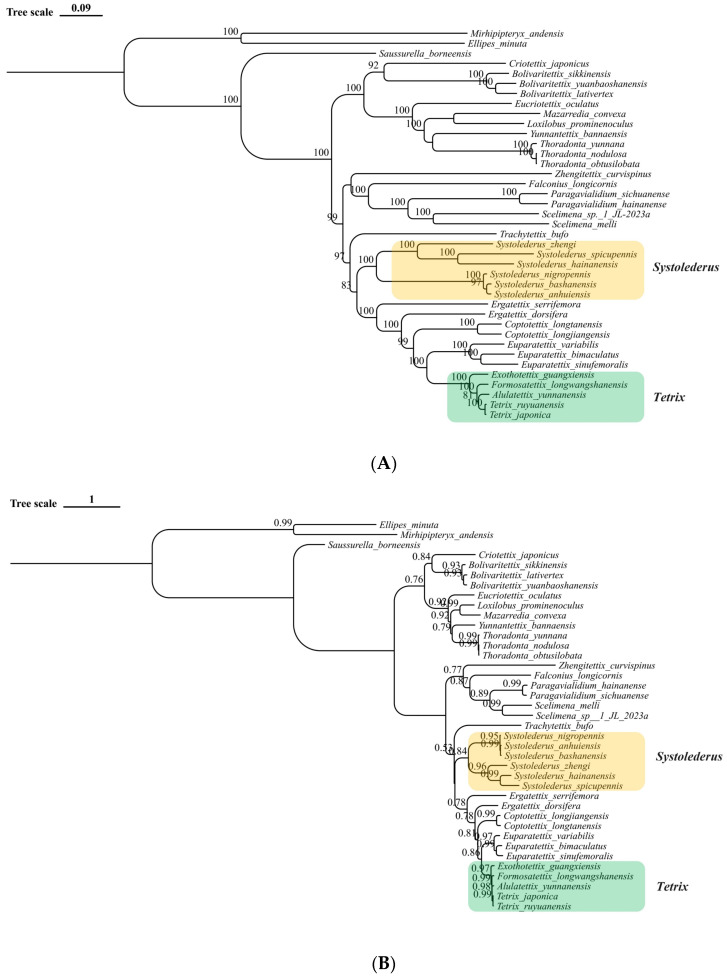
Phylogenetic trees using the PCGs in all 39 currently available mitogenomes of Tetrigidae. (**A**) The maximum likelihood mitogenomic tree. The numbers on each node represent the bootstrapping values. (**B**) The Bayesian Monte Carlo Markov Chain (MCMC) mitogenomic tree. The numbers on each node represent the posterior possibility values.

We conducted a phylogenetic analysis using the 11,056 bp concatenated nucleotide alignment of all the protein-coding genes. Remarkably, the generated maximum likelihood (ML) tree and the Bayesian Monte Carlo Markov Chain (MCMC) tree possess an analogous topology, corresponding with the established morphological classification schema, with species of the same genus assembling into discrete clusters (Figure 6A,B). Our integrative phylogenetic reconstruction, predicated on the trees above, discloses engaging biological insights into the classification and evolutionary liaisons among several Tetrigidae species. The tree compellingly delineates multiple clades demonstrating high bootstrap support scores, indicating clade solidity and confidence in their clustering (Figure 6A,B).

A salient cluster comprising *Ex. guangxiensis*, *F. longwangshanensis*, and species of *Tetrix* raises significant interest. Both *Ex. guangxiensis* and *F. longwangshanensis* show a close association with *Tetrix ruyuanensis* and *T. japonica*, which is indicative of a common evolutionary ancestor. The positioning of *Eu. sinufemoralis* is undoubtedly well-defined as an independent subclade in the present tree. Remarkably, our study refrains from exclusively classifying *Ex. guangxiensis* and *F. longwangshanensis* as separate species under the *Tetrix* generic group, following their short branch length and monophyly. Given their peripheral position within the clade, their evolutionary relationship may reflect recent liaison among extremely close species. As such, we boldly surmise that this categorization does not corroborate their pre-established separate-species status and is the over-division of the *Tetrix* genus caused by rapid morphological adaptive evolution.

The interrelation involving the merged *Systolederus* generic group warrants particular attention. All selected *Systolederus* spp. share a single ancestry, as indicated by their monophyly positioning within the tree, but in two independent subclades. The conspecific systematic status of *Systolederus* is not undisputed. Our results indicate that this genus is heterogeneous and should be split. Our results suggest this genus can be generally divided into at least two geographical species complexes. We observed a strongly supported subclade with exceptionally short branch lengths, reflecting a species group distributed along the Yangtze River basin. *S. bashanensis*, *S. anhuiensis*, and *S. nigropennis* formulate a subclade within this assemblage. Notably, the branch length for this subclade is reasonably shorter than the subclade that is constituted by *S. zhengi*, *S. hainanensis,* and *Systolederus spicupennis*, implying a substantial divergent evolutionary pattern within this genus. This subclade links populations from Shaanxi and Sichuan (*S. bashanensis*), and Jiangsu & and Anhui (*S. anhuiensis*), provinces, showing a close phylogenetic affinity between these geographical locations.

### 3.5. Divergence Dating

**Figure 7:**
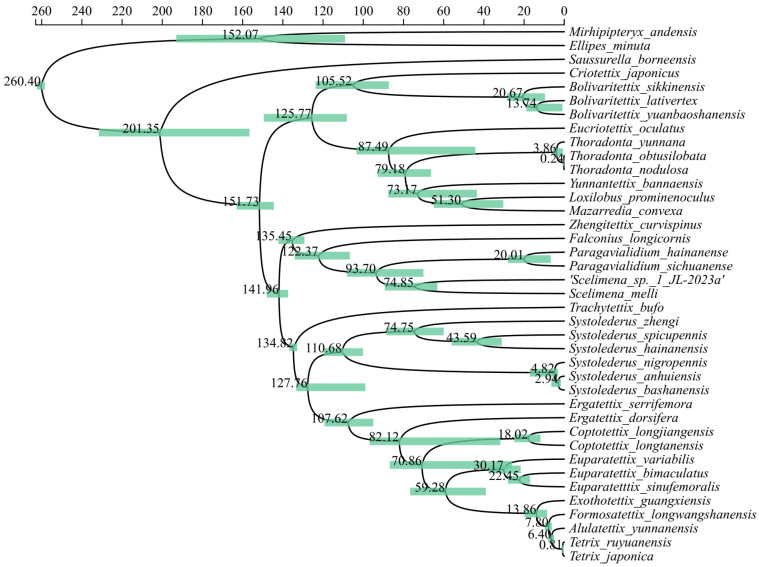
The phylogenetic analysis based on the mitogenomic data with calibrated divergence times within the Tetrigidae family utilizing the Bayesian Evolutionary Analysis Sampling Trees (BEAST) methodology (scale represented in Mya). Each node number denotes the estimated time of divergence, with the pale green bars illustrating the span of the estimated value ranges.

In this study, we conducted divergence time estimations for the Tetrigidae family with two calibration points (Figure 7). Such assessment considerably enriches our understanding of the divergence history among the species represented in the generated phylogenetic tree. Remarkably, the calibrated time trees displayed identical topologies, in total conformity with the previously developed maximum likelihood (ML) and Bayesian inference (BI) trees. This consistency underlines the robustness of our alignment and mitochondrial phylogenomic methodologies.

The illuminated divergence time estimations illustrate the fragmentation process of the Tetrigidae family, affirming that it was initiated over 150 million years ago (Mya), around 151.73 Mya. This finding aligns with widely accepted scientific perspectives that categorize this family among the most ancient lineages within the Orthoptera order [31,32]. This study also detected considerable variability in the timings of differentiation across diverse genera within this family. The explicit genus *Bolivaritettix*, for instance, embarked on a divergence journey between 13.74 and 20.67 Mya. Conversely, the genus *Scelimena*’s divergence is postulated to have occurred around 74.85 Mya. Notably, the divergence timescales of the five *Tetrix*-like species of our primary interest spanned between 0.81 and 13.86 Mya. Although these times parallel the observed divergence times amongst other taxa, they embody a higher granularity, evoking a distinct gradient in the differentiation process. The temporal disjunction between *Eu. sinufemoralis* and *Eu. bimaculatus* constitutes a definitive proof of their classification as a particular species, with their divergence timeframe at 22.45 Mya (Figure 7).

Significant differences in divergence times were revealed between two distinct subclades within the genus *Systolederus*. The combined divergence of these two subclades approaches 110.68 Mya, nearing the estimated split of the entire Tetrigidae family. This observation suggests that *Systolederus* is the oldest genus within Tetrigidae, or that it has resulted from heterogeneously merged multiple lineages that diverged anciently. In our opinion, further subdivision and reorganization are required to capture the marked divergence intervals more precisely and refine our understanding of the phylogenetic patterns in this group. Additionally, two subclades were noticed to have distinct divergence patterns. Analysis showed the divergence period between the first subclade, comprising *S. zhengi*, *S. spicupennis,* and *S. hainanensis*, and the second clade extends far back in time, to approximately 74.75 Mya. In stark contrast, the results for the subclade containing *S. nigropennis*, *S. anhuiensis,* and *S. bashanensis* indicated a relatively recent split, estimated at only 2.94 to 4.82 Mya (Figure 7), which suggests that they may have been established by a rapid geological range expansion and should be split into an independent species group or even a new genus.

**Figure 8:**
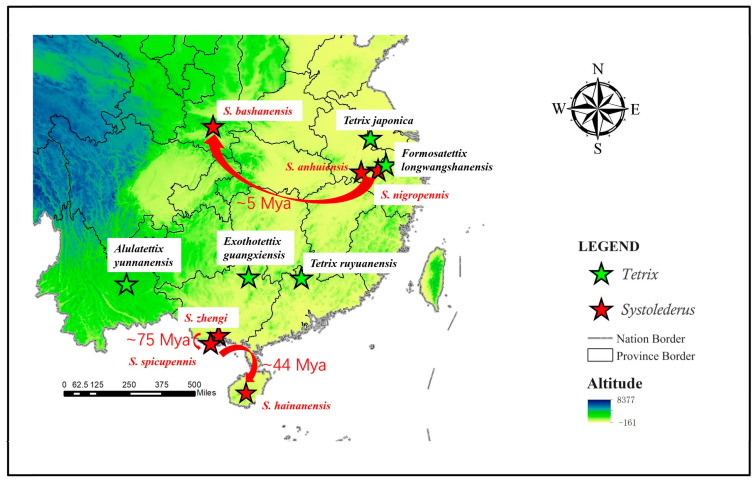
The geographic locations of the sample collection points for the *Systolederus* and *Tetrix* genera. The red stars represent the sampling locations for species within the *Systolederus* genus. The green stars denote sampling points for the *Tetrix* genus. The map depicts a region of China, with red arrows indicating the inferred diffusion spread of the *Systolederus* genus constructed from the time divergence tree.

To further investigate the unique evolutionary patterns of species within the *Systolederus* and *Tetrix* generic groups, we mapped the sample collection locations of taxa on the time-divergence tree (Figure 8). We found that the two subclades of *Systolederus* are distributed in the southern and northern regions, respectively, with great geographical distance between them and highly divergent temporal ordering. Specifically, the southern subclade exhibits close geographical proximity among the three species, with overlapping distributions in Guangxi province, yet a considerable divergence time estimated at approximately 75 Mya. In contrast, the northern species span over 500 km between sampling locations but reflect less than 5 million years of divergence time. These results support the existence of distinct northern and southern diffusion–divergence patterns within *Systolederus*.

The *Tetrix* generic group, on the other hand, displayed a notably widespread geographical distribution, forming its current broad distribution across an evolutionary timescale estimated at only ~14 Mya for our combined clade—far shorter than the other genera despite the vast geographical range. This suggests that this genus has evolved rapid dispersal capabilities, allowing for the colonization of diverse habitats over short evolutionary periods.

## 4. Discussion

The burgeoning field of Tetrigidae phylogenetics has its cornerstone in morphology, which forms the basis of an intricate and complex systematic comprising over 100 genera to date [1,9,11]. In this study, we introduced complementary hypotheses to this well-established morphological framework, expecting that integrating additional molecular marker sequences could elucidate minor discrepancies in the current morphological classification system; some morphological features may lead to blurred taxonomic boundaries. We contributed the complete mitochondrial genomes of four contentious species, and, by integrating these with the existing mitogenome data from other Tetrigidae species, we constructed a revised phylogenetic tree and estimated their divergence times. Although limited in scale, our findings aid in refining the morphological classification system of Tetrigidae, with a specific emphasis on the delineation between the *Systolederus* and *Tetrix* generic groups. We highlight differential evolutionary patterns that may confound morphological classifications and offer insights for more comprehensive future molecular systematic studies.

Through the comparative mitochondrial genomic analysis of the four new species’ records with the previously published mitogenomes, our study yielded primarily two insights: the current genus *Systolederus* is a monophyletic assembly that warrants division; conversely, *Tetrix*, a widespread generic group, may be over-split. The narrow fastigium of *Systolederus*, while characteristic enough to suggest a clade, does not withstand as a decisive morphological feature when considering the substantial divergence between at least two subclades and the distribution ranges that far exceed what is typically circumscribed within a single genus; it would be more accurate to split these species into a generic group instead of a genus. With some differentiation events in the southern species dating back to ~75 Mya, incorporating the many early-diverging lineages within the *Systolederus* generic group at a subfamily level is plausible. Our findings are consistent with the latest reviews, which contend that the species richness of the *Systolederus* generic group is unwarrantedly high and geographically overextended, suggesting an urgent need to reassess this genus with additional molecular evidence [11,14]. Moreover, our data suggest that the trait of a narrow fastigium is an ancient one, potentially a plesiomorphic characteristic, preserved as a vestige rather than favored by natural selection. This needs to be further verified with more extensive molecular data.

In stark contrast, as hypothesized, the Tetrix generic group appears to exhibit signs of excessive splitting [15,16,17]. The provided mitochondrial data for *Ex. guangxiensis*, *F. longwangshanensis*, and *Alulatettix yunnanensis* position these species within a short-branch monophyletic clade with *Tetrix*, characterized by a relatively recent diversification within the Tetrigidae’s phylogeny. Structure analysis has already implied that these species share one specific potential tRNA-Ile, which provides a compelling case for reclassifying these taxa, as the same evolutionary event might have shaped them. These species’ geographical distributions and high-altitude niches imply natural solid selection pressures, which may have contributed to adaptive diversifications. Thus, even though typical species like *T. japonica* and *T. ruyuanensis* anchor this clade, its evolutionary novelty persuades us to widen the taxonomic boundaries of the *Tetrix* generic group. The distinctiveness of these species may be attributed to adaptive radiation, and thus, a reassessment is warranted, involving more molecular evidence, particularly from the nuclear genome [6,7,8,15].

In conclusion, our results prompt a reevaluation of the taxonomic limits at the genus level for generic groups like *Systolederus* and *Tetrix* based on mitogenomes, highlighting the necessity for extensive molecular evidence to correctly resolve the taxonomic boundaries which divergent patterns of trait evolution can confound. This study’s mitochondrial markers, combined with our systematic phylogenetic evaluation, divergence time estimations, and synthesis of prior research, propose a division of the *Systolederus* generic group and a conflation within the *Tetrix* generic group. While we acknowledge the preliminary nature of our work, we believe that it lays the groundwork for subsequent in-depth molecular phylogenetic research on these genera and the Tetrigidae family at large.

## Data Availability

The raw sequencing data for the four pygmy grasshoppers’ mitogenomes were uploaded to the Figshare database under the DOI:10.6084/m9.figshare.24849756.

## Supplementary Materials

The following supporting information can be downloaded at: https://www.mdpi.com/article/10.3390/insects15030174/s1, Supplement Table S1: The nucleotide composition, AT, and GC skew statistics of the four sequenced mitogenomes. Supplement Table S2: Genbank accessions for mitogenomes used in this study. Supplement Table S3: Codon usage analysis of the protein-coding genes in the mitogenomes used in this study.
